# ART26.12, a novel fatty acid‐binding protein 5 inhibitor, shows efficacy in multiple preclinical neuropathy models

**DOI:** 10.1002/ejp.4718

**Published:** 2024-08-26

**Authors:** W. G. Warren, M. Osborn, A. David‐Pereira, C. Tsantoulas, Wenwen Xue, A. Yates, S. E. OSullivan

**Affiliations:** ^1^ Artelo Biosciences Ltd., Alderley Park Alderley Edge Cheshire UK; ^2^ Transpharmation Ltd. The London Bioscience Innovation Centre London UK; ^3^ Pharmaron Inc. Beijing People's Republic of China

## Abstract

**Background:**

Painful neuropathy is a pathological condition caused by numerous factors including diabetes, chemotherapy or cancer. ART26.12 is a novel fatty acid‐binding protein 5 inhibitor, which our group showed could prevent and treat persistent pain in a preclinical model of oxaliplatin‐induced peripheral neuropathy.

**Methods:**

In the current study, the efficacy of orally dosed ART26.12 was tested in multiple neuropathy models of different aetiology. Paw withdrawal threshold to von Frey monofilaments and latency to escape a cold plate were used as measurements of mechanical and cold sensitivity.

**Results:**

ART26.12 (25 and 50 mg/kg BID), dosed prior to the induction of paclitaxel‐induced peripheral neuropathy (PIPN), reversed mechanical allodynia induced by paclitaxel in both male and female rats, and ART26.12 (50 mg/kg BID) prevented the induction of PIPN in female rats. ART26.12 (50 mg/kg BID) also had a protective effect on body weight in the PIPN model. ART26.12 (25 and 100 mg/kg BID) reversed mechanical allodynia when treating established streptozotocin‐induced diabetic neuropathy in male rats. In a model of breast cancer‐induced bone pain in female rats, ART26.12 (100 mg/kg BID) reversed mechanical allodynia within 1 h of dosing. In the same model, ART26.12 (25 mg/kg BID) reversed mechanical allodynia from day 4 of treatment.

**Conclusion:**

Overall, these preclinical data suggest that ART26.12 is a safe and efficacious therapeutic drug for continued development towards the prevention and treatment of peripheral neuropathy.

**Significance Statement:**

This work now shows that ART26.12, a novel and selective inhibitor of FABP5, can prevent and treat multiple preclinical models of peripheral neuropathy. Given its excellent safety profile, further work is warranted to develop ART26.12 as a potential therapeutic tool for pain management.

## 1 INTRODUCTION

Fatty acid‐binding protein (FABP)5 is 1 of 10 human FABP isoforms; a group of proteins that are responsible for fatty acid transport, metabolism and signalling (Kaczocha et al., 2009, 2012; Sanson et al., 2014). FABP5 is known to transport endocannabinoids (eCBs) and other *N*‐acylethanolamines (NAEs), which are implicated in pain signalling via cannabinoid receptor type 1 (CB_1_) and transient receptor potential cation channel subfamily V member 1 (TRPV1) (Berger et al., 2012; Clapper et al., 2010; Richardson et al., 1998). Research has shown that inhibition or genetic knockout of FABP5 has anti‐nociceptive effects in several models of pain, including inflammatory pain, osteoarthritis and chronic constriction injury (Berger et al., 2012; Bogdan et al., 2018, 2022; Fauzan et al., 2022; Gordon et al., 2024; Kaczocha et al., 2014, 2015; Peng et al., 2017; Yan et al., 2018; Zhu et al., 2023). We recently showed that the novel FABP5 inhibitor ART26.12 prevented and treated oxaliplatin‐induced peripheral neuropathy (OIPN) in a CB_1_‐dependent manner, with involvement from TRPV1, CB_2_ and peroxisome proliferator‐activated receptor alpha (Warren et al., 2024). This also coincided with widespread lipid modulation, especially *N*‐acyl amino acids. The anti‐nociceptive effects of FABP5 inhibition appear to be mediated by reduced transport of anandamide and other NAEs to catabolic enzymes such as fatty acid amide hydrolase (Berger et al., 2012; Kaczocha et al., 2009, 2014, 2015). This causes an increase in eCB and NAE tone, which activates cannabinoid targets, producing an analgesic effect. ART26.12 is minimally brain penetrant, and its actions appear to be mediated through the peripheral nervous system.

Medical cannabis and cannabis‐based medicines have been used to treat chronic neuropathic pain in humans (Petzke et al., 2022). Several systematic reviews of randomized controlled trials have evaluated the efficacy of these medications with mixed results. While some suggest that cannabis may be a useful treatment for neuropathic pain (Andreae et al., 2015; Dykukha et al., 2021; Petzke et al., 2016), others have warned of potential safety issues regarding central nervous system (CNS) effects (Mücke et al., 2018). However, activation of the cannabinoid targets CB_1_, CB_2_ and TRPV1 leads to analgesia in many forms of pain (Kaczocha et al., 2014). Peripheral FABP inhibition may provide a novel strategy for targeting the eCB system while avoiding CNS side effects. ART26.12 was shown to be safe and tolerable with no observed adverse effect limit of 1000 mg/kg in rats and dogs (Warren et al., 2024).

Having previously shown ART26.12 to be efficacious in OIPN, the aim of this study was to assess ART26.12 in peripheral neuropathies of different causes. We assessed the efficacy of ART26.12 in paclitaxel‐induced peripheral neuropathy (PIPN), streptozotocin (STZ)‐induced diabetic neuropathy and cancer‐induced bone pain, where cannabinoid receptor engagement is known to be efficacious (Deng, Cornett, et al., 2015; Deng, Guindon, et al., 2015; Gonçalves et al., 2022; Lozano‐Ondoua et al., 2010, 2013).

## 2 METHODS

### 2.1 Animals

Animals were housed in Perspex cages in groups of four in a controlled environment of constant temperature and moisture (temperature: 21 ± 1°C, light:dark cycle of 12:12 h). Food and water were available ad libitum. Observations for clinical signs of tolerability were made daily throughout the duration of studies. PIPN and STZ experiments were carried out according to the Animals (Scientific Procedures) Act, 1986 (U.K. Government Home Office). The sex, species and weight of animals used in each experiment are listed in the appropriate experimental sub‐sections below. These studies were performed under the Home Office project licence number PPL‐708841. The cancer‐induced bone pain study was performed according to guidelines approved by the Institutional Animal Care and Use Committee (IACUC) of Pharmaron following the guidance of the Association for Assessment and Accreditation of Laboratory Animal Care (AAALAC). All experiments conformed to the ARRIVE guidelines.

### 2.2 Chemicals

ART26.12 (Charnwood Molecular) was dissolved in 5% DMSO and 20% vitamin E d‐α‐tocopherol polyethylene glycol 1000 succinate in deionized water to produce a volume of 5 mL/kg. Duloxetine (Kemprotec) was dissolved in deionized water to produce a volume of 5 mL/kg. Paclitaxel (Pac; Sigma‐Aldrich) was sonicated in 5% ethanol and 5% Kolliphor EL solution to produce a volume of 5 mL/kg. The formulation was prepared fresh on the day of dosing. STZ (Sigma) was dissolved in 20 mM citrate buffer to make a volume of 10 mL/kg. Tramadol was dissolved in saline to produce a volume of 5 mL/kg.

### 2.3 Apparatus and experimental procedure

Mechanical allodynia was assessed using calibrated von Frey filaments or an electronic von Frey instrument (Bioseb SAS) applied to the plantar surface of the left hind paw. Calibrated filaments were used in the PIPN and STZ models, while the electronic instrument was used in the cancer‐induced bone pain experiment. The animals' paw withdrawal threshold (PWT) was measured in grams using Dixon's up‐down method (Chaplan et al., 1994; Dixon, 1980). Withdrawal behaviours were characterized by immediate flinching to the stimulus. Cold allodynia was assessed using a cold plate (Bioseb SAS), which was maintained at 15°C. Latency to the first escape behaviour was used as the outcome measure. Escape behaviours were characterized as paw licking, withdrawal or jumping. All measurements were taken by investigators blinded to the treatment group.

### 2.4 Paclitaxel‐induced peripheral neuropathy

**FIGURE 1:**
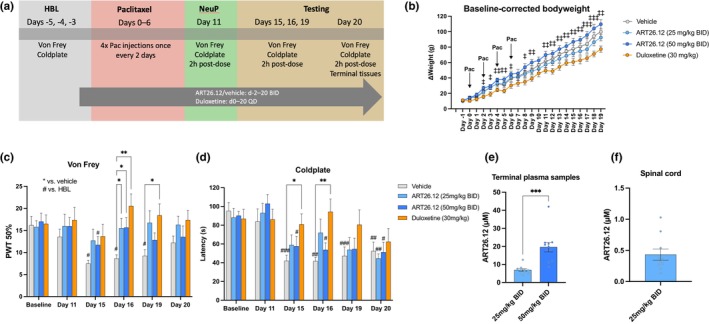
The effects of ART26.12 on paclitaxel‐induced peripheral neuropathy (PIPN) in male rats. PIPN was induced by four doses of Pac given IP on Days 0, 2, 4 and 6. ART26.12 and vehicle were administered PO, BID from Day −2 onwards, while duloxetine was administered PO, QD from Day 0 onwards (a). Body weight measurements were taken from Days −1 to 19 (b). von Frey 50% paw withdrawal thresholds and cold plate latencies were taken on Days 11, 15, 16, 19 and 20 (c, d). Terminal plasma and spinal cord samples were taken on Day 20 (e, f). (b), (c) and (d) used two‐way repeated measures analyses of variance. (e) used an unpaired *t*‐test with Welch correction for unequal standard deviation. *p < 0.05, **p < 0.01, ***p < 0.001 indicate significant reversal of mechanical allodynia or cold hyperalgesia by treatment when compared to the respective groups' vehicle values. ^#^ *p* < 0.05, ^##^ *p* < 0.01, ^###^ *p* < 0.001 indicate significant decrease in von Frey threshold or cold plate latency when compared to the respective groups' baseline values. ^‡^ *p* < 0.05, ^‡‡^ *p* < 0.01, ^‡‡‡^ *p* < 0.001 indicate significant difference in baseline‐corrected body weight between ART26.12 (50 mg/kg) and duloxetine. Vehicle (*n* = 10); ART26.12 (25 mg/kg BID; *n* = 10); ART26.12 (50 mg/kg BID; *n* = 11); duloxetine (*n* = 10). BID, bi‐daily; IP, intraperitoneal; Pac, paclitaxel; PO, oral; PWT, paw withdrawal threshold; QD, once daily.

**FIGURE 2:**
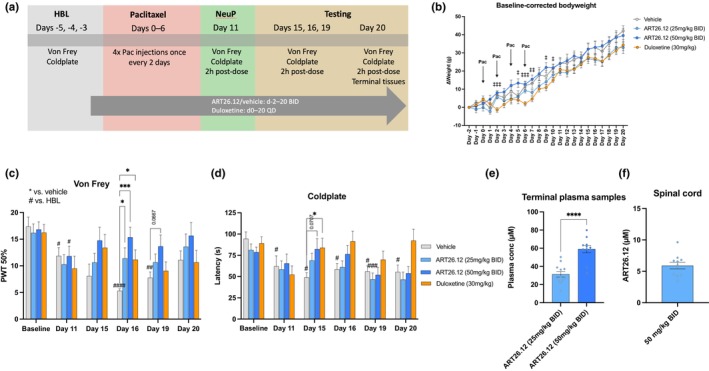
The effects of ART26.12 on paclitaxel‐induced peripheral neuropathy (PIPN) in female rats. PIPN was induced by four doses of Pac given IP on Days 0, 2, 4 and 6. ART26.12 and vehicle were administered PO, BID from Day −2 onwards, while duloxetine was administered PO, QD from Day 0 onwards (a). Body weight measurements were taken from Day −1 to 19 (b). von Frey 50% paw withdrawal thresholds and cold plate latencies were taken on Days 11, 15, 16, 19 and 20 (c, d). Terminal plasma and spinal cord samples were taken on Day 20 (e, f). (b), (c) and (d) used two‐way repeated measures analyses of variance. (e) used an unpaired *t*‐test with Welch correction for unequal standard deviation. *p < 0.05, ***p < 0.001, ****p < 0.0001 indicate significant reversal of mechanical allodynia or cold hyperalgesia by treatment when compared to the respective groups' vehicle values. ^#^ *p* < 0.05, ^##^ *p* < 0.01, ^###^ *p* < 0.001, ^####^ *p* < 0.0001 indicate significant decrease in von Frey threshold or cold plate latency when compared to the respective groups' baseline values. ^‡^ *p* < 0.05, ^‡‡^ *p* < 0.01, ^‡‡‡^ *p* < 0.001 indicate significant difference in baseline‐corrected body weight between ART26.12 (50 mg/kg) and duloxetine. Vehicle (*n* = 11); ART26.12 (25 mg/kg BID; *n* = 12); ART26.12 (50 mg/kg BID; *n* = 11); duloxetine (*n* = 12). BID, bi‐daily; IP, intraperitoneal; Pac, paclitaxel; PO, oral; PWT, paw withdrawal threshold; QD, once daily.

This study used 48 adult female and 48 adult male Sprague–Dawley rats (180–210 g; Charles River; for study schematic, see Figures 1a and 2a). Following at least 1 week of acclimatization, PWT and cold plate latency were assessed on 3 consecutive days (Days −5, −4 and −3). The mean of Day −4 and Day −3 was considered the healthy baseline (HBL) prior to PIPN induction. Animals were ranked and randomized according to their PWT values. On Day −2, bi‐daily (BID; 8:00 AM and 4:00 PM) oral (PO) treatment with vehicle or ART26.12 (25 or 50 mg/kg BID) began. On Days 0, 2, 4 and 6, Pac (2 mg/kg/dose) was administered intraperitoneally (IP). On Day 0, daily (QD; 8:00 AM) PO administration of duloxetine was initiated. After the final Pac injection, animals recovered for 4 days. Body weight was measured daily throughout the experiment. On Days 11, 15, 16, 19 and 20, PWT thresholds and cold plate latencies were assessed 2 h after the 8:00 AM doses of vehicle, ART26.12 or duloxetine. On Day 20, mechanical and cold sensitivity were assessed 2 h after the final dose of the test compound. Immediately after the last behavioural measurement on Day 20, animals were culled by CO_2_ overdose. Terminal blood samples were taken via cardiac puncture. Whole spinal cords and L4–L6 dorsal root ganglia were collected from the vehicle and ART26.12 groups.

### 2.5 Streptozotocin‐induced diabetic neuropathy

**FIGURE 3:**
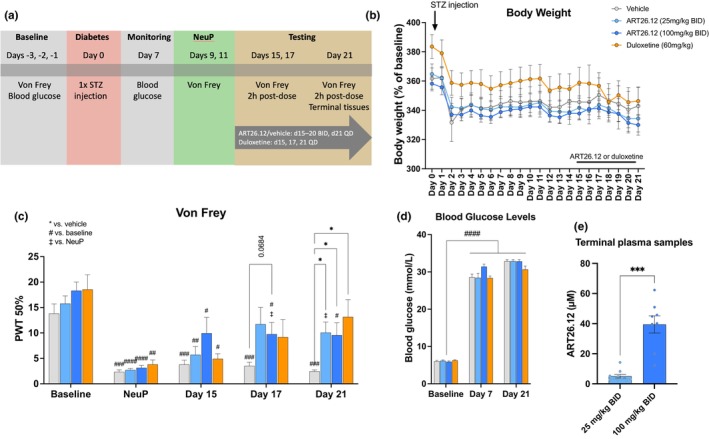
The effects of ART26.12 on diabetic neuropathy in male rats. Diabetic neuropathy was induced by streptozotocin (STZ) given IP on Day 0. ART26.12 and vehicle were administered PO, BID from Day 15 to 20 and QD on Day 21 and duloxetine was administered PO, QD on Days 17, 19 and 21 (a). Body weight measurements were taken from Day 0 to 21 (b). von Frey measurements were taken on Days −3, −2, −1 (baseline), 9 and 11 (NeuP) and 15, 17 and 21 of treatment (c). Blood glucose levels were measured on Days 0, 7 and 21 (d). Terminal plasma samples were taken on Day 21 (e). (b), (c) and (d) used two‐way repeated measures analyses of variance. (e) used an unpaired *t*‐test with Welch correction for unequal standard deviation. *p < 0.05, ***p < 0.001 indicates significant reversal of mechanical allodynia by treatment when compared to the respective groups’ vehicle values. ^‡^ *p* < 0.05 indicates a significant increase in PWT when compared with the respective groups NeuP. ^#^ *p* < 0.05, ^##^ *p* < 0.01, ^###^ *p* < 0.001, ^####^ *p* < 0.0001 indicate a significant decrease in von Frey threshold or increase in blood glucose when compared to the respective groups' baseline values. Vehicle (*n* = 9); ART26.12 (25 mg/kg BID; *n* = 10); ART26.12 (100 mg/kg BID; *n* = 9); duloxetine (*n* = 7). BID, bi‐daily; IP, intraperitoneal; NeuP, neuropathic baseline; PWT, paw withdrawal threshold; PO, oral; QD, once daily.

This study used 52 adult male Wistar rats (325–350 g; Envigo; for study schematic, see Figure 3a). Animals were housed in groups of two and acclimatized to the laboratory environment for at least 1 week. Prior to STZ injection, PWT was assessed on 3 consecutive days (Days −3, −2, and −1). The mean of Day −2 and Day −1 was considered the HBL. Animals were injected IP with STZ (55 mg/kg) on Day 0. Body weights were monitored daily throughout the experiment. On Day 7, blood glucose levels were measured in one drop of blood using an Exactive Vital (Microtec Medical) blood glucose monitor. Only STZ‐injected rats with blood glucose concentrations above 16 mmol/L were considered diabetic and included in the study. A terminal blood glucose concentration was measured at the end of the study. von Frey readings were taken on Day 9 and Day 11 post‐STZ treatment to confirm development of mechanical allodynia. The average of the two readings was taken as the neuropathic baseline (NeuP). Animals were then ranked and randomized according to their percentage change in PWT values. ART26.12 (25 or 100 mg/kg) or vehicle was given PO on Days 15–20 (BID) and Day 21 (QD). Duloxetine (60 mg/kg) was given PO on test days only. von Frey thresholds were assessed on Days 15, 17 and 21 at 2 h post‐dosing. Two hours after the last behavioural measurement on Day 21, animals were culled by CO_2_ overdose. Terminal blood samples were taken via cardiac puncture.

### 2.6 Cancer‐induced bone pain

**FIGURE 4:**
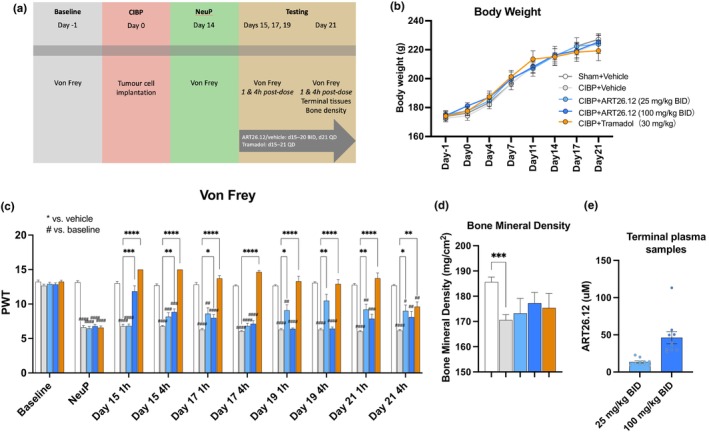
The effects of ART26.12 on cancer‐induced bone pain (CIBP) in female rats. CIBP was induced by injection of breast cancer cells given IP on Day 0. ART26.12 and vehicle were administered PO, BID from Day 15–20 and QD on Day 21, and tramadol was administered PO, QD on Day 15–21 (a). Body weight measurements were taken from Days −1, 0, 4, 7, 11, 14, 17 and 21 (b). Electronic von Frey measurements were taken on Days −1 and 14, as well as 1 and 4 h post‐dosing on Days 15, 17, 19 and 21 (c). Bone mineral density was measured on Day 21 (d). Terminal plasma samples were taken on Day 21 (e). *p < 0.05, **p < 0.01, ***p < 0.001, ****p < 0.0001 indicate significant reversal of mechanical allodynia by treatment when compared to the respective groups' CIBP vehicle values. ^#^ *p* < 0.05, ^##^ *p* < 0.01, ^###^ *p* < 0.001, ^####^ *p* < 0.0001 indicate a significant decrease in von Frey threshold when compared to sham–vehicle groups baseline values. Vehicle (*n* = 10); ART26.12 (25 mg/kg BID; *n* = 10); ART26.12 (100 mg/kg BID; *n* = 10); tramadol (*n* = 10). BID, bi‐daily; IP, intraperitoneal; NeuP, neuropathic baseline; PWT, paw withdrawal thresholds; PO, oral; QD, once daily.

This study used 50 female adult Sprague–Dawley rats (162–190 g; Beijing Vital River; for study schematic, see Figure 4a). PWT was assessed on Day −1. On Day 0, animals were anaesthetized, the left tibia was carefully exposed and a gauge needle was inserted in the intramedullary canal of the bone. Around 50,000 murine breast cancer cells in a PBS vehicle were then injected into the tibial bone cavity. PWT readings were taken on Day 14 post‐breast cancer cell injection to provide the NeuP. ART26.12 (25 or 100 mg/kg BID) or vehicle was given PO on Days 15–21. The positive control, tramadol (30 mg/kg QD) was given IP on Days 15–21. PWT was assessed on Days 15, 17, 19 and 21 at 1 and 4 h post‐dosing. Following anaesthetization on Day 21, left tibial bone density measurements and blood samples were taken, after which animals were culled.

### 2.7 Data analysis

Data were analysed using a two‐way repeated measures analysis of variance with time and treatment as the within‐ and between‐subject factors respectively. This was followed by Dunnett's post hoc test (treatment groups vs. vehicle, HBL or NeuP) for PWT and latency data, and Tukey's post hoc test for body weight data (Prism, GraphPad Software). Data in all experiments are expressed as mean ± SEM.

In the STZ model, 17 animals dosed with STZ did not remain above the diabetic blood glucose threshold (16 mmol/L) and were excluded from analysis. Two animals that did not reach the 50% PWT reduction threshold were excluded. Two further animals that did not maintain the 50% PWT reduction on neuropathy days were removed using the interquartile range outlier test. Final numbers (*n*) for the STZ study were vehicle (*n* = 9); ART26.12 (25 mg/kg BID; *n* = 10); ART26.12 (100 mg/kg BID; *n* = 9); and duloxetine (*n* = 7).

## 3 RESULTS

### 3.1 Paclitaxel‐induced peripheral neuropathy (male cohort)

Animals treated with ART26.12 (50 mg/kg BID) showed a greater increase in body weight than those treated with duloxetine on most days (*p* < 0.05–0.001; Figure 1b).

Vehicle PWTs were significantly lower than HBL on Day 15 (*p* = 0.0189), 16 (*p* = 0.0465) and 19 (*p* = 0.0414), demonstrating the establishment of mechanical allodynia. Animals treated with ART26.12 (25 mg/kg BID) and duloxetine did not show a reduction in PWT at any time point compared to HBL. Animals treated with the higher dose of ART26.12 (50 mg/kg BID) only showed lower PWT scores than HBL at Day 15 (*p* = 0.0131). When compared with vehicle, ART26.12 (25 and 50 mg/kg BID) reversed mechanical allodynia by increasing PWT scores on Day 16 (*p* = 0.0350 and 0.0309, respectively), and duloxetine increased PWT scores on Day 16 (*p* = 0.0043) and Day 19 (*p* = 0.0201; Figure 1c).

Vehicle cold plate latencies were significantly lower than HBL on Days 15 (*p* = 0.0005), 16 (*p* = 0.0026), 19 (*p* = 0.0001) and 20 (*p* = 0.0038), demonstrating the establishment of cold hyperalgesia. Animals treated with ART26.12 (25 mg/kg BID) only showed significantly lower latency scores on Day 20 (*p* = 0.0057) when compared with HBL. At 50 mg/kg BID, animals treated with ART26.12 displayed cold allodynia on Day 15 (*p* = 0.0498), 16 (*p* = 0.0211) and 20 (*p* = 0.0107). When compared with vehicle, duloxetine reversed cold allodynia by increasing latency scores on Day 15 (*p* = 0.0213) and Day 16 (*p* = 0.0095; Figure 1d).

Terminal plasma concentrations of ART26.12 (25 and 50 mg/kg BID) were 6.9 ± 0.7 and 19.6 ± 2.7 μM, respectively (Figure 1e). An unpaired *t*‐test with Welch correction for unequal standard deviations showed that plasma concentrations in the ART26.12 (50 mg/kg BID) group were significantly higher than the ART26.12 (25 mg/kg BID) group (*t*(11.52) = 4.591, *p* = 0.0007). Spinal cord concentrations of ART26.12 (25 mg/kg) were 0.4 ± 0.1 μM (Figure 1f).

### 3.2 Paclitaxel‐induced peripheral neuropathy (female cohort)

On Days 2, 5, 6, 7, 9 and 10, animals treated with vehicle or ART26.12 (25 or 50 mg/kg BID) showed a greater increase in body weight than duloxetine (*p* < 0.05–0.0001; Figure 2b).

In female rats, PWTs in the vehicle group were significantly different from HBL on Day 11 (*p* = 0.0313), Day 16 (*p* < 0.0001) and Day 19 (*p* = 0.0028), with a strong trend towards significance on Day 15 (*p* = 0.0529). Animals treated with ART26.12 (25 mg/kg BID) and duloxetine did not show a reduction in PWT at any time point compared to HBL values within their groups. ART26.12 (50 mg/kg BID) only showed lower PWT scores than HBL on Day 11 (*p* = 0.0276). When compared with vehicle, ART26.12 (25 mg/kg BID; *p* = 0.0281), ART26.12 (50 mg/kg BID; *p* = 0.0008) and duloxetine (*p* = 0.0248) increased PWT scores on Day 16. ART26.12 (50 mg/kg BID) also trended towards reversal of mechanical allodynia on Day 19 (*p* = 0.0667; Figure 2c).

Vehicle cold plate latencies showed a small but significant reduction from HBL on Day 15 (*p* = 0.0114), Day 16 (*p* = 0.0262), Day 19 (*p* = 0.0177) and Day 20 (*p* = 0.0168). None of the treatment groups showed a significant reduction in latency when compared with HBL, apart from ART26.12 (25 mg/kg BID) on Day 19 (*p* = 0.0002). When compared with vehicle on Day 15, duloxetine (*p* = 0.0353) reversed cold allodynia by increasing cold plate latency, while ART26.12 (50 mg/kg BID; *p* = 0.0707) trended towards reversal (Figure 2d).

Plasma concentrations of ART26.12 (25 and 50 mg/kg BID) were 31.3 ± 3.1 μM and 59.1 ± 4 μM, respectively (Figure 2e). An unpaired *t*‐test with Welch correction for unequal standard deviations showed that plasma concentrations in the ART26.12 (50 mg/kg BID) group were significantly higher than the ART26.12 (25 mg/kg BID) group (*t*(19.09) = 5.480, *p* < 0.0001). Spinal cord concentrations of ART26.12 (50 mg/kg BID) were 5.9 ± 0.5 μM (Figure 2f).

### 3.3 Streptozotocin‐induced diabetic neuropathy

Injection of STZ caused a sharp drop in body weight in all animals. However, there were no significant differences in body weight between the treatment groups (Figure 3b).

PWT scores in the vehicle (*p* = 0.0005), ART26.12 (25 mg/kg BID; *p* < 0.0001), ART26.12 (50 mg/kg BID; *p* < 0.0001) and duloxetine (*p* = 0.0021) groups were significantly lower at the NeuP when compared with HBL, demonstrating the induction of neuropathy. Animals treated with ART26.12 (25 mg/kg BID) and duloxetine did not show significantly reduced PWTs on Days 17 and 21 (days 3 and 7 of drug treatment) when compared with HBL, indicating attenuation of allodynia. When compared with NeuP, ART26.12 (25 and 100 mg/kg BID) increased PWT scores on Days 21 (*p* = 0.0202) and 17 (*p* = 0.0272) respectively. On Day 17, ART26.12 (100 mg/kg BID) showed a trend towards reversal of mechanical allodynia (*p* = 0.0684). On Day 21, ART26.12 (25 mg/kg BID; *p* = 0.0134), ART26.12 (100 mg/kg BID; *p* = 0.0475) and duloxetine (*p* = 0.0476) significantly increased PWT scores when compared with vehicle (Figure 3c).

Blood glucose levels were elevated on Day 7 and Day 21 in all treatment groups (*p* < 0.0001; Figure 3d). Mean plasma concentrations for ART26.12 (25 and 100 mg/kg BID) were 5.1 ± 1.2 and 39.3 ± 5.7 μM, respectively (Figure 3e). An unpaired *t*‐test with Welch correction for unequal standard deviations showed that plasma concentrations in the ART26.12 (100 mg/kg BID) group were significantly higher than the ART26.12 (25 mg/kg BID) group (*t*(7.57) = 5.879, *p* = 0.0005).

### 3.4 Cancer‐induced bone pain

There were no significant differences in body weight between treatment groups (Figure 4b).

All groups, except the sham‐vehicle group, showed a reduction in PWT at NeuP when compared with HBL (*p* < 0.0001). On Day 15 (day 1 of drug treatment), ART26.12 (100 mg/kg BID) and tramadol reversed mechanical hyperalgesia by increasing PWTs at 1 h (*p* = 0.0003 and *p* < 0.0001) and 4 h (*p* = 0.0031 and *p* < 0.0001) post‐dosing when compared with vehicle. On Day 17, ART26.12 (100 mg/kg BID) and tramadol increased PWT scores at 1 h post‐dose (*p* = 0.0261 and *p* < 0.0001), and tramadol increased PWT scores at 4 h post‐dose (*p* < 0.0001). On Day 19, ART26.12 (25 mg/kg BID) and tramadol increased PWTs at 1 h (*p* = 0.0217 and *p* < 0.0001) and 4 h (*p* = 0.0046 and *p* < 0.0001; Figure 4g). On Day 21, ART26.12 (25 mg/kg BID) and tramadol increased PWTs at 1 h (*p* = 0.0070 and *p* < 0.0001) and 4 h (*p* = 0.0264 and *p* < 0.01; Figure 4c).

There were no significant differences in bone mineral density between treatment groups (Figure 4d). Mean plasma concentrations for ART26.12 (25 and 100 mg/kg BID) were 13.5 ± 1.48 and 46.25 ± 8.12 μM, respectively (Figure 4e). An unpaired *t*‐test with Welch correction for unequal standard deviations showed that plasma concentrations in the ART26.12 (100 mg/kg BID) group were significantly higher than the ART26.12 (25 mg/kg BID) group (*t*(9.59) = 3.696, *p* = 0.0029).

## 4 DISCUSSION

ART26.12 is a selective inhibitor of FABP5 that has previously shown efficacy in the prevention and treatment of preclinical OIPN (Warren et al., 2024). This study assessed whether ART26.12 is effective in other preclinical models of peripheral neuropathy. These included the prevention of PIPN and the treatment of diabetic neuropathy and cancer‐induced bone pain. In the PIPN prevention model, ART26.12 reversed mechanical allodynia and attenuated cold allodynia in both sexes. In the treatment of established diabetic neuropathy, ART26.12 also reversed STZ‐induced mechanical allodynia. When treating breast cancer‐induced bone pain, ART26.12 showed dose‐/time‐dependent effects where the high dose (100 mg/kg BID) was efficacious immediately and the lower dose (25 mg/kg BID) reversed mechanical allodynia after a few days of dosing. These results, coupled with our previous study (Warren et al., 2024), suggest that ART26.12 may be a promising and safe treatment for use across peripheral neuropathies of diverse aetiology.

We previously showed that ART26.12 effectively treated and prevented OIPN (Warren et al., 2024). The current results now show that ART26.12 is also efficacious in the prevention of PIPN at the same doses. This was best observed on Day 15/16 when PIPN‐induced neuropathy was most pronounced in the vehicle‐treated groups in both male and female animals. This effect was not seen towards the end of the experiment as vehicle groups began to show natural resolution of mechanical allodynia. As observed in OIPN, the effects of ART26.12 were more consistent in mechanical than cold sensitivity. Together, this suggests that ART26.12 is a potential therapy for CIPN caused by different types of chemotherapy agents. Males treated with the low dose of ART26.12 (25 mg/kg BID) showed a marginally better response than those treated with the high dose (50 mg/kg BID), while females showed a greater response to the higher dose. On Day 16, ART26.12 was also more effective at reducing mechanical allodynia than duloxetine in females, which may have been due to reduced efficacy of duloxetine in the female cohort. Previous research has shown that oestrogen deficiency in females can attenuate the anti‐depressive effects of duloxetine (Xu et al., 2017). These sex differences do not seem to be attributable to ART26.12 plasma exposure. This is in accordance with our previous study, which showed that elevated ART26.12 in the plasma was not linked to increased efficacy (Warren et al., 2024). Female rats showed a 3‐ to 4.5‐fold increase in ART26.12 plasma exposure when comparing equivalent doses with males, suggesting there are sex differences in the metabolism of ART26.12 (although this may be specific to rats). Drug exposure differences, coupled with known sex differences in the eCB system (ART26.12's putative mechanism of action), may influence the optimal analgesic dose in females compared with males (Blanton et al., 2021).

To examine the broader potential of ART26.12 in peripheral neuropathy, we next examined the potential of ART26.12 to be utilized as a treatment therapy in models of diabetic neuropathy and cancer‐induced bone pain. As with CIPN, we found that ART26.12 reversed mechanical allodynia in both models by increasing PWT when compared with vehicle‐treated animals, and the effects persisted for at least 4 h post‐dosing. In both models, the reversal of mechanical allodynia was observed after 3 or 4 days of dosing with 25 mg/kg ART26.12. Our previous work in the treatment of OIPN also showed a similar effect where 7 days of dosing with ART26.12 (25 mg/kg BID) caused a twofold improvement in PWT when comparing the first and last doses (Warren et al., 2024). In diabetic neuropathy, ART26.12 dosing significantly reversed PWT by Day 3, while duloxetine was only effective after 7 days of dosing. In the cancer‐induced bone pain model, the higher dose of ART26.12 (100 mg/kg BID) showed immediate reversal of mechanical allodynia after dose 1, with a reduction in efficacy as the study continued. Downstream activation of CB_1_ with the higher dose may cause receptor downregulation, a common effect of CB_1_ agonists (Piscura et al., 2023), although this development of tolerance was not observed with 25 mg/kg BID. Interestingly, the efficacy of tramadol in this model also decreased with repeated dosing.

Our previous OIPN study showed that ART26.12 primarily exerted its analgesic effect via CB_1_, with involvement from CB_2_, TRPV1 and PPARα (Warren et al., 2024). While antagonist experiments and lipidomics were not conducted in the current study, it is likely that ART26.12 mediated its effects in these neuropathic pain models through similar mechanisms. Other cannabinoid agonists have produced CB_1_‐dependent analgesia in platinum‐based CIPN models (Bagher et al., 2023; Deng, Cornett, et al., 2015; Deng, Guindon, et al., 2015; Mulpuri et al., 2018; Pascual et al., 2005; Rahn et al., 2014; Sepulveda et al., 2022; Vera et al., 2013). Similarly, CB_1_‐ and CB_2_‐dependent analgesia has been shown in diabetic neuropathy and cancer‐induced bone pain, as well as PIPN (Deng et al., 2012; Gonçalves et al., 2022; Lin et al., 2022; Lozano‐Ondoua et al., 2010, 2013; Toniolo et al., 2022; Xu et al., 2014; Zeng et al., 2024; Zhang et al., 2018). TRPV1 and the wider eCB system are therapeutic targets in these pathologies (de Almeida et al., 2021; Liu et al., 2023; Rossato et al., 2018; Sun et al., 2019; Thompson et al., 2020; Xie et al., 2022; Zhang et al., 2019). Finally, *N*‐arachidonoyl‐glycine, a lipid previously shown to be elevated by ART26.12 (Warren et al., 2024), showed analgesic effects in a preclinical neuropathy model (Vuong et al., 2008). While these studies demonstrate a common mechanism of action across different neuropathies, activation of the eCB system also mediates analgesia in a wide variety of pain models. For example, FABP5 inhibition or knockdown produces CB_1_‐dependent analgesia in inflammatory pain, osteoarthritis and chronic constriction injury (Berger et al., 2012; Bogdan et al., 2018, 2022; Fauzan et al., 2022; Gordon et al., 2024; Kaczocha et al., 2014, 2015; Peng et al., 2017; Yan et al., 2018; Zhu et al., 2023). Overall, this suggests that ART26.12 may also have therapeutic potential in chronic pain conditions with an inflammatory component.

In conclusion, ART26.12 successfully prevented CIPN caused by paclitaxel, and treated painful symptoms of diabetic neuropathy and cancer‐induced bone pain. ART26.12 is effective in both males and females. Given its favourable safety profile and efficacy, ART26.12 is an exciting prospect as an orally active, peripherally selective, non‐opioid, non‐steroidal analgesic for the treatment of persistent painful conditions.

## AUTHOR CONTRIBUTIONS

This study was designed by S.E.O. and A.Y. The experiments were performed by A.D.‐P. and C.T. The data were analysed by W.G.W. and M.O. and the results were critically examined by all authors. W.G.W. had a primary role in preparing the manuscript, which was edited by S.E.O., A.Y. and M.O. All authors have approved the final version of the manuscript and agree to be accountable for all aspects of the work.

## CONFLICT OF INTEREST STATEMENT

This research was fully funded by Artelo Biosciences. WGW, MO, AY and SOS are paid employees of Artelo Biosciences.
